# Nilotinib related acute myocardial infarction with nonobstructive coronary arteries: a case report and literature review

**DOI:** 10.1186/s12872-022-02504-0

**Published:** 2022-02-13

**Authors:** Weiwei Chen, Beibei Du, Kun Liu, Zhixi Yu, Xingtong Wang, Ping Yang

**Affiliations:** 1 Department of Cardiology, China-Japan Union Hospital of Jilin University, Xiantai, Street No. 126, Changchun, 130033 Jilin Province China; 2 Jilin Provincial Cardiovascular Research Institute, Jilin Provincial Engineering Laboratory for Endothelial Function and Genetic Diagnosis of Cardiovascular Disease, Changchun, 130031 Jilin Province China; 3 Department of Hematology, The First Hospital of Jilin University, Jilin Provincial Hematology Research Institute, National Key Discipline in Hematology and Oncology, Changchun, 130021 Jilin Province China

**Keywords:** Myocardial Ischemia with No Obstructive Coronary Artery Disease, Coronary artery spasm, Nilotinib, Ergonovine provocation test, Vascular adverse events, Case report

## Abstract

**Background:**

Myocardial Ischemia with No Obstructive Coronary Artery Disease (MINOCA) is a common cause of type 2 acute myocardial infarction (AMI) which requires careful differential diagnosis. Coronary artery spasm (CAS) syndrome is one etiology that can lead to MINOCA. Nilotinib, a targeted treatment for chronic myeloid leukemia (CML), has been reported to be related with increased risk of adverse vascular events.

**Case presentation:**

A 67-year-old male patient was admitted to hospital with acute chest pain. He had a past medical history of CML and a history of treatment with nilotinib for 12 months. Coronary angiography (CAG) showed no significant stenosis. Since the onset of angina was generally in the early morning, and ECG and echocardiography suggested right coronary artery (RCA) disease, an ergonovine provocation test was performed to confirm the diagnosis of CAS. After intracoronary administration of ergonovine, middle and distal RCA showed over 90% vasoconstriction. Nilotinib related MINOCA, CAS and CML were diagnosed. Lifestyle changes (cessation of smoking), anti-spasmodics, statin treatment and adjustment of the nilotinib dose (from 200 mg bid, to 150 mg bid) were recommended for this patient. Six-month’s follow-up showed good recovery with no onsets of angina.

**Conclusions:**

Physicians should be vigilant to adverse vascular events when treating patients who have been prescribed nilotinib. It is suggested that in patients with MINOCA who have a history of treatment with nilotinib, CAS-induced MINOCA should be included in the differential diagnosis. Further studies are needed to clarify the mechanism and to find better management.

## Background

The increased understanding of acute myocardial infarction (AMI) has led to the need for a general, worldwide definition. The European Society of Cardiology (ESC), the American College of Cardiology (ACC) and the American Heart Association (AHA), together with the World Heart Federation (WHF), brought out the Fourth Universal Definition of myocardial infarction (MI) in 2018, and reclassified AMI into 5 subtypes[1]. Any ischemic myocardial injury that is caused by the imbalance of oxygen supply and oxygen need was classified as type 2 MI [1]. Myocardial ischemia with no obstructive coronary artery disease (MINOCA) commonly leads to type 2 MI which needs careful differential diagnosis [2]. The diagnosis of MINOCA should be made in the absence of obstructive (lesion ≥ 50%) coronary artery disease. Coronary artery spasm (CAS) syndrome, coronary dissection, in situ thrombosis [3], tachycardia, and coronary embolism [4] are all etiologies that can cause MINOCA. The careful collection of medical history, and specialized examinations such as intracoronary imaging or the ergonovine provocation test, can facilitate the differential diagnosis.

Nilotinib, a second-generation BCR-ABL tyrosine kinase inhibitor (TKI), has greatly improved the treatment and prognosis of chronic myeloid leukemia (CML) [5]. Adverse vascular events (including angina, type 1 MI, and peripheral artery diseases) have been reported in patients treated with nilotinib [6, 7]. Here we report the diagnosis and treatment of a rare case of nilotinib related MINOCA.

## Case presentation

A 67-year-old male patient was admitted to China-Japan Union hospital for persistent chest pain two days before admission in Aug. 2020. The patient’s chest pain started 2 days previously with no inducement, and was gradually alleviated 30 min after oral nitroglycerin intake. Over the next two days before he was referred to hospital, he had onsets of angina in the early mornings. The patient was diagnosed with CML 6 years previously. The patient was initially treated with imatinib, which was then changed to nilotinib (300 mg *bid*) in Dec 2019. A cytogenetic and molecular response was achieved in Mar 2020. The patient had no history of hypertension or diabetes, but had a 45-pack-year smoking history. No abnormalities were found during physical examinations.

Cardiac injury biomarkers showed significantly increased levels of troponin I (4.4 ng/ml; normal range 0–0.05), myoglobin (181 mg/L; normal range 0–107), and CK-MB (49.0 U/L; normal range 0–4.3). D-dimer and NT-proBNP levels were slightly increased. LDL-C level was 3.41 mmol/L, and blood sugar level was normal. Other laboratory tests were within the normal range (Detailed information see Table 1).

**Table 1:** Laboratory test results of this patient

Category	Value [Normal range]
White blood cell (*10^9^/L)	8.4 [4–10]
Neut (%)	80↑ [50–70]
Troponin I (ng/ml)	4.4 ↑ [0–0.05]
Myoglobin (mg/L)	181↑ [0–107]
CK-MB (U/L)	49.0↑ [0–4.3]
D-dimer (ng/ml)	837 ↑ [0–600]
NT-pro BNP	540 ↑ [300–450]
LDL-C (mmol/L)	3.41↑ [< 1.8]
Other tests	Normal

**Fig. 1:**
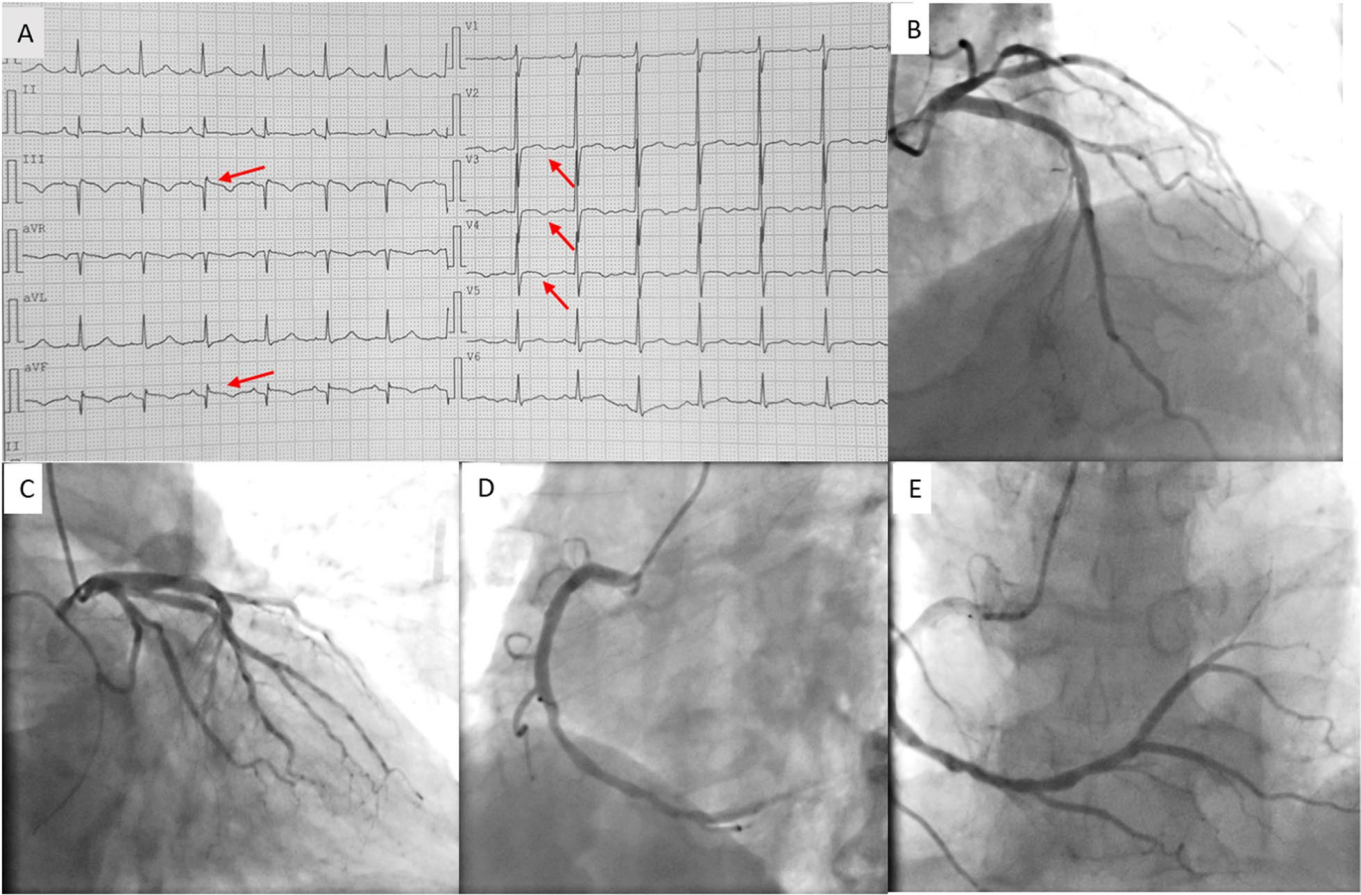
ECG upon admission, and Baseline CAG. **A** Admission ECG: Leads III and aVF: pathological Q waves, slightly elevated ST segments. Precordial leads: T wave inversions. Red arrows indicate abnormalities in ECG. **B**–**E**. Baseline CAG. CAG showed ~ 30-40% stenosis in pLAD (**B**) and pOM (**C**) 30–40%stenosis in dRCA (**D**, **E**). ECG: electrocardiography, CAG: coronary angiography, pLAD: proximal left anterior descending artery, pOM: proximal obtuse marginal artery, dRCA: distal right coronary artery

**Fig. 2:**
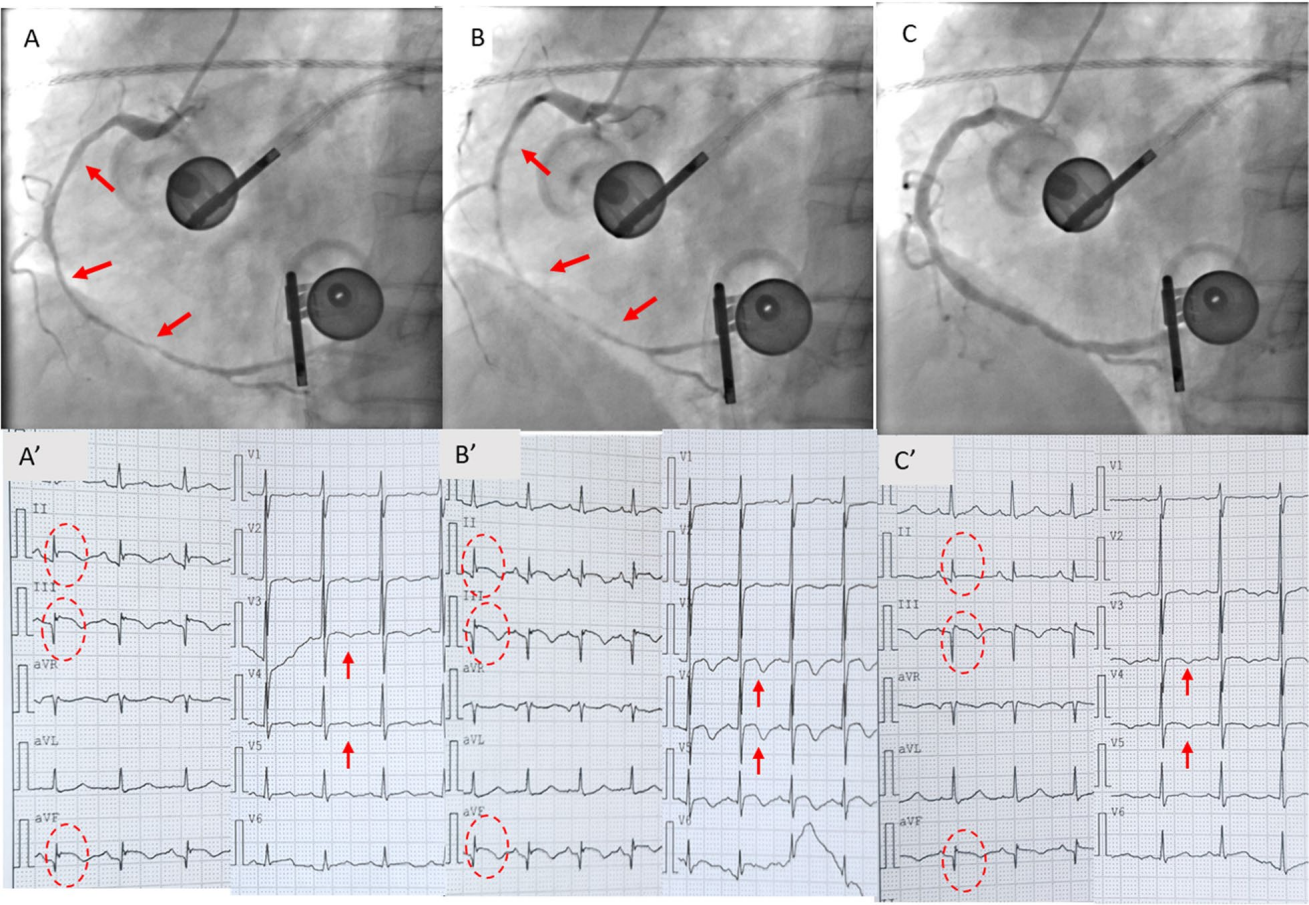
Serial CAG and ECG during ergonovine provocation test. **A**, **A’** ECG and CAG 2 min after intracoronary administration of intracoronary administration of ergonovine. Middle and distal segments of RCA showed diffuse coronary spasm, ≥ 90% diffuse stenosis in middle and distal RCA. ECG showed dynamic changes in lead II, III and aVF (ST elevation) and precordial leads (T wave inversion). **B**, **B’** ECG and CAG 5 min after intracoronary administration of ergonovine. Middle and distal segments of RCA showed diffuse coronary spasm, nearly subtotal occlusion in middle and distal RCA. ECG showed dynamic changes in lead II, III and aVF (ST elevation) and precordial leads (T wave inversion). **C**, **C’** ECG and CAG after intracoronary administration of nitroglycerin. Middle and distal segments of RCA quickly returned to normal. ECG also returned to baseline. ECG: electrocardiography, CAG: coronary angiography, RCA: right coronary artery

Electrocardiography (ECG) upon admission showed pathological Q waves and slightly elevated ST segments in Leads III and aVF. T wave inversions were also found in precordial leads (Fig. 1A). ECG showed inferior wall hypokinesis and normal cardiac function (EF 60%). Holter monitoring for 24 h showed no ventricular arrhythmias (VAs). Color doppler ultrasound of carotid and subclavian arteries were normal. Coronary angiography (CAG) was performed and showed approximately 30%-40% stenosis in the proximal left anterior descending artery (pLAD) and the proximal obtuse marginal artery (pOM) (Figs. 1B, C). There was only mild to moderate stenosis (30–40%) in the anticipated culprit vessel right coronary artery (RCA; Fig. 1D, E). Since there had been frequent onsets of angina in the early mornings, and also no significant or suspicious lesions were found during CAG, coronary spasm was considered the etiology. An ergonovine provocation test was performed to confirm the diagnosis of CAS. After intracoronary administration of ergonovine (0.04 mg), the middle and distal segments of RCA showed diffuse coronary spasm (Fig. 2A, B) compared to the baseline CAG of RCA (Fig. 1D, E). 5 min after ergonovine was administered, CAG showed near subtotal occlusion in middle and distal RCA. Continuous ECG monitoring showed more prominent ST elevations in inferior wall leads (Fig. 2A’, B’). The patient also had chest discomfort after ergonovine administration, which later developed into chest pain at 5 min. The ECG showed dynamic changes, which were all rapidly relieved after intracoronary administration of nitroglycerin (Fig. 2C, C’).

According to the medical history, the CAG findings, and the ergonovine provocation test, the patient was diagnosed with Nilotinib related MINOCA, Killip class I, CAS syndrome, and CML.

Lifestyle changes were recommended including the cessation of smoking. Stenting was not recommended as there was no severe stenosis and after ergonovine administration diffuse spasm were observed. Intensified anti-spasmodics were recommended for long-term management with Isoket ampoule injection (10 mg/h IV) and Diltiazem Sustained Release Tablets (90 mg *bid*). Statins were also prescribed to maintain the stability of the plaque and to protect the endothelial function. After hematologist consultation, the nilotinib dose was adjusted to 150 mg *bid*.

The chest pain was completely relieved by the above treatments. The only adjustment to the treatment schedule occurred five days later with the replacement of the Isoket ampoule injection with Isoladine 50 mg once daily (QD). The patient was discharged one week after the procedure. Follow-ups were conducted every three months, and after six months there was still good recovery with no onsets of angina. Also during follow-ups, no peripheral artery rapid progression or new cerebrovascular disease were found.

## Discussion

Here we presented a rare case of nilotinib related MINOCA, and further confirmed that CAS was the etiology of MINOCA, using an ergonovine provocation test. Symptom relief and recovery were achieved by lifestyle changes, anti-spasmodic medications, and adjustment of the nilotinib dose.

MINOCA has been shown to account for 5–15% of AMI cases [2, 8]. Furthermore, as a “working” but not a final diagnosis, screening for the underlying etiology of MINOCA is vital for subsequent proper management. Careful collection of medical history and specialized examinations, are required in the differential diagnosis [8]. For this patient, the history of nilotinib treatment is important in the exploration of the underlying etiology.

BCR-ABL tyrosine kinase inhibitors (TKIs) have shifted the treatment paradigm, and significantly improved the prognosis of chronic myeloid leukemia (CML). However, in recent years, vasculotoxicity or vascular adverse events (VAE) (including peripheral arteries and coronary artery) have become a concern in patients receiving such treatment [9]. Although not fully characterized, based on three retrospective studies [9–11], patients who developed TKI-related VAE commonly had their first VAE onset within the first year. For this patient, he started the medication (nilotinib) since Dec. 2019, and had AMI 9 months (Aug. 2020) afterward. This is also consistent to the results of the retrospective studies.

Compared to the first generation BCR-ABL TKI (imatinib), the risk of VAE with nilotinib (second generation BCR-ABL TKI) was over threefold higher [11]. In one pooled analysis of three prospective clinical studies, during a quite long follow-up of 77 months in 108 patients who were treated with nilotinib, the incidence of angina/MI was 6% (7/108) [10]. Apart from AMI, other potent VAE such as cerebrovascular or peripheral artery rapid progression, which manifested as intermittent claudication or stroke, have also been reported [7, 12, 13]. Still, the diagnosis is mainly made based on medical history and clinical experience.

The mechanism of nilotinib related cardiovascular disease is still unclear. Lipid metabolism disorder [14], endothelial dysfunction [9] and inflammation overactivation [15] are several suggested mechanisms, which can accelerate atherosclerotic process and cause vascular spasm [16]. To the best of our knowledge, there have been only three cases reported of nilotinib related coronary spasm [17, 18]. No CAS induced MINOCA related to nilotinib has been previously reported.

Patients with traditional cardiovascular (CV) risk factors, such as old age (≥ 60 years), smoking, or high cholesterol level, were at higher risk of developing vasculotoxicities [19]. Therefore, baseline systematic coronary risk evaluation (SCORE) assessments and baseline CML-CV scores were developed to enable the prediction of adverse CV events [20]. For this patient, advanced age, smoking and high cholesterol level are all risk factors in developing vasculotoxicity. So, during the CAG, atherosclerosis and coronary spasm should also be assessed.

Focal or diffuse coronary spasm can show concomitant changes with plaque rupture/erosion in obstructive AMI patients (Type I AMI). CAS can be a common cause of MINOCA and an ergonovine/ acetylcholine provocation test is the gold standard for the diagnosis of CAS. An ergonovine provocation test was implemented by evaluating the response of intracoronary administration of ergonovine/ acetylcholine by CAG. CAS was defined as > 90% vasoconstriction of an epicardial coronary artery and compromised coronary blood flow. Although ergonovine/ acetylcholine provocation test is considered the gold standard for diagnosis, it is not routinely used in most catheter labs in China due to safety concerns; this can lead to misdiagnosis and under realization of CAS. In one study, the percentage of CAS diagnosed with the positive provocation test was 46.2% (37/80) and also in this study, good safety data with no major adverse events were reported [21]**.**

The manifestations of CAS can vary vastly from being asymptomatic to angina, AMI, ventricular arrhythmias (VAs), or even syncope, with different coronary segments involved and different frequencies of angina onsets [22]. The treatments of CAS mainly involves lifestyle changes (such as cessation of smoking), anti-spasmodic medications, and other cardiovascular protective therapy [22]. Stenting for diffuse coronary spasm is not recommended, but in patients with focal spasm, its utility is unclear [22–24]. In patients with frequent onsets of angina, risk stratification is needed and for high-risk patients with life-threatening VAs, ICD therapy is recommended for secondary prevention [24]. For this patient, with above optimized medical treatment, symptom relief and good recovery were also achieved.

Optimal management of nilotinib related CAD needs the cooperation and teamwork of cardiologists and hematologists, especially in those with life-threatening MI. Specialized examinations such as intracoronary imaging [25] or ergonovine testing can facilitate the differential diagnosis, which can help to make proper treatment and guarantee better prognosis.

## Conclusions

Physicians should be vigilant to adverse vascular events when treating patients who have been prescribed nilotinib. For MINOCA patients with a history of nilotinib treatment, an ergonovine provocation test should be done to check for the presence of CAS induced MINOCA. Further studies are needed to clarify the mechanism and to find better management options.

## Data Availability

The data/figures used and/or analyzed in this case are available from the corresponding author upon reasonable request.

## Abbreviations

AMI: Acute myocardial infarction
CAS: Coronary artery spasm
CAG: Coronary angiography
CML: Chronic myeloid leukemia
MINOCA: Myocardial Ischemia with No Obstructive Coronary Artery Disease
RCA: Right coronary artery
TKI: Tyrosine kinase inhibitor
